# The effect of job stress appraisals on mental health among health professionals: The mediating role of work engagement

**DOI:** 10.1192/j.eurpsy.2021.1244

**Published:** 2021-08-13

**Authors:** A. Chaabouni

**Affiliations:** Department Of Psychology And Pedagogic Science, St Mary’s University, Twickenham, United Kingdom

**Keywords:** health professionals, work engagement, Depression, Anxiety

## Abstract

**Introduction:**

Health professionals face an increased risk of developing mental health difficulties due to work-related stress. It has been demonstrated that work engagement has a protective role on mental health from work-related stress. The majority of the research on the psychological impact of job stress among health professionals focused on the work-related stressors or the type of stressors as challenges or hindrances. However, the impact might depend on an individual’s appraisal of challenges and hindrances.

**Objectives:**

- Examine the effects of job appraisals on mental health. - Establish the role of work engagement as a mediator between them.

**Methods:**

An online survey was completed by 196 health professionals and included questionnaires about job appraisals, stressors (variety of tasks, responsibility and cooperation with colleagues), work engagement, anxiety and depression.

**Results:**

Appraising stressors as challenges did not have any direct impact on mental health, whereas hindrance appraisals had a negative influence. Participants who appraised cooperation with colleagues as challenging reported lower levels of depression through higher work engagement (B = − 0.17, 95% CI [− 0.354, − 0.027]). Appraising variety of tasks as a hindrance predicted higher levels of depression through lower work engagement (B = 0.150, 95% CI [0.041, 0.289]). Participants appraising the other two stressors as hindrances were more anxious and depressed through lower work engagement.

**Conclusions:**

The negative psychological impact of hindrance appraisals was persistent, whereas the positive impact of challenge appraisals through work engagement depended on the stressor. Stress interventions may need to consider both the type of appraisal and the type of stressor.

